# Validation of the MAGGIC (Meta-Analysis Global Group in Chronic Heart Failure) heart failure risk score and the effect of adding natriuretic peptide for predicting mortality after discharge in hospitalized patients with heart failure

**DOI:** 10.1371/journal.pone.0206380

**Published:** 2018-11-28

**Authors:** Sayma Sabrina Khanam, Eunhee Choi, Jung-Woo Son, Jun-Won Lee, Young Jin Youn, Junghan Yoon, Seung-Hwan Lee, Jang-Young Kim, Sung Gyun Ahn, Min-Soo Ahn, Seok-Min Kang, Sang Hong Baek, Eun-Seok Jeon, Jae-Joong Kim, Myeong-Chan Cho, Shung Chull Chae, Byung-Hee Oh, Dong-Ju Choi, Byung-Su Yoo

**Affiliations:** 1 Department of Cardiology, Wonju College of Medicine, Yonsei University, Wonju, Republic of Korea; 2 Smith Center for Outcomes Research in Cardiology, Beth Israel Deaconess Medical Center, Harvard Medical School, Boston, MA, United States of America; 3 Yonsei University College of Medicine, Seoul, Korea; 4 The Catholic University of Korea, Seoul, Korea; 5 Sungkyunkwan University College of Medicine, Seoul, Korea; 6 University of Ulsan College of Medicine, Seoul, Korea; 7 Chungbuk National University College of Medicine, Cheongju, Korea; 8 Kyungpook National University College of Medicine, Daegu, Korea; 9 Department of Internal Medicine, Seoul National University Hospital, Seoul, Korea; 10 Seoul National University Bundang Hospital, Seongnam, Korea; Institut d'Investigacions Biomediques de Barcelona, SPAIN

## Abstract

**Background:**

In clinical practice, a risk prediction model is an effective solitary program to predict prognosis in particular patient groups. B-type natriuretic peptide (BNP)and N-terminal pro-b-type natriuretic peptide (NT-proBNP) are widely recognized outcome-predicting factors for patients with heart failure (HF).This study derived external validation of a risk score to predict 1-year mortality after discharge in hospitalized patients with HF using the Meta-analysis Global Group in Chronic Heart Failure (MAGGIC)program data. We also assessed the effect of adding BNP or NT-proBNP to this risk score model in a Korean HF registry population.

**Method and results:**

We included 5625 patients from the Korean acute heart failure registry (KorAHF) and excluded those who died in hospital. The MAGGIC constructed a risk score to predict mortality in patients with HF by using 13 routinely available patient characteristics (age, gender, diabetes, chronic obstructive pulmonary disorder (COPD), HF diagnosed within the last 18 months, current smoker, NYHA class, use of beta blocker, ACEI or ARB, body mass index, systolic blood pressure, creatinine, and EF). We added BNP or NT-proBNP, which are the most important biomarkers, to the MAGGIC risk scoring system in patients with HF. The outcome measure was 1-year mortality. In multivariable analysis, BNP or NT-proBNP independently predicted death. The risk score was significantly varied between alive and dead groups (30.61 ± 6.32 vs. 24.80 ± 6.81, p < 0.001). After the conjoint use of BNP or NT-proBNP and MAGGIC risk score in patients with HF, a significant difference in risk score was noted (31.23 ± 6.46 vs. 25.25 ± 6.96, p < 0.001).The discrimination abilities of the risk score model with and without biomarker showed minimal improvement (C index of 0.734 for MAGGIC risk score and 0.736 for MAGGIC risk score plus BNP or NT-proBNP, p = 0.0502) and the calibration was found good. However, we achieved a significant improvement in net reclassification and integrated discrimination for mortality (NRI of 33.4%,p < 0.0001 and IDI of 0.002, p < 0.0001).

**Conclusion:**

In the KorAHF, the MAGGIC project HF risk score performed well in a large nationwide contemporary external validation cohort. Furthermore, the addition of BNP or NT-proBNPto the MAGGIC risk score was beneficial in predicting more death in hospitalized patients with HF.

## Introduction

Heart failure (HF) imposes a great health problem worldwide. The associated risks of HF are particularly vulnerable in countries with aging populations where diagnosis, treatment, and prevention of re-hospitalization are difficult [1]. Hospitalized patients with HF have poor prognosis, but re-hospitalization rates are increasingly, consequently elevating the in-hospital and post-discharge mortality rates. The prognosis of HF remains poor, and repeated hospitalization exerts a huge cost on national health care budgets and threatens quality of life[2–5]. Therefore, taking urgent measures to predict a patient’s clinical course as early as possible is necessary by selecting evidence-based management strategies to improve care of patients with HF. To date, risk stratification in these patients is a challenge [6].

Risk prediction models are frequently used to triage patients and ease treatment decisions, helping physicians estimate prognosis in a more neutral manner and interpret the consequence of prognosis studies[7, 8].Among all the datasets, the most eminently prognostic information could facilitate appropriate implication of monitoring and treatment that improves the standard of nursing and outcomes of hospitalized patients with HF[7]. Several predictors are applied in a prediction model to assess the risk of occurrence of a specified event (death, re-hospitalization) in the future[9].

Recently, the Meta-analysis Global Group in Chronic Heart Failure (MAGGIC) has performed a literature-based meta-analysis by collecting each patient data from 30studies including six randomized clinical trials. These data consist of important prognostic variables, such as demographics, medical history, medical treatment, symptom status, clinical variables, laboratory variables, and outcome[10,11]. Hence, a risk score was established to predict mortality in patients with HF from 13 regularly available patient characteristics at a baseline level, and an easily accessible online calculator was made available (www.heartfailurerisk.org).Tracing risk stratification depicts a prospective outcome and is beneficial for patients with advanced HF (AHF) of multiple etiologies. Meanwhile, risk modeling serves as a comprehensive and conducive assessment for clinicians and plays a facilitating role to patients and health service provider to achieve better recognition of likely outcomes[12].MAGGIC also provides an advantage for determining numerous individual risk factors, such as serum sodium, gender, and survival of patients with HF with preserved or reduced LVEF[13–15].However, B-type natriuretic peptide(BNP), a widely recognized outcome-predicting factor for patients with HF, has not been included in a risk prediction model, as it was only available in less than 25% of the enrolled patients[16, 17].BNP and N-terminal pro-b-type natriuretic peptide (NT-proBNP) are valuable prognostic predictors for grading severities and predicting mortality risk in patients with HF[18,19]. Measurement of serum BNP is essential in guiding decision-making process and predicting patient status, as well as in establishing therapeutic strategies[20–22]. Thus, considering a major risk factor in a risk scoring system is necessary to predict important outcome. Although BNP and NT-pro BNP are of accurate prognosis-predictive ability for hospitalized patients with HF, other clinical factors may also play pivotal roles in affecting outcomes [23].

An established, simple, cost-effective model with the highest accuracy, which consists of selective number of predictors, is significant for use in other populations[8]. MAGGIC risk score in HF was previously conducted and validated in a Swedish population, and an excellent discrimination was obtained [9]. Nevertheless, the real-world performance of prediction models for new patient populations and original advancing target populations including Asians is not consistent[24]. Therefore, in this study, we aimed to derive and externally validate a risk score to predict 1-year mortality after discharge in hospitalized patients with HF using the MAGGIC program data. We also assessed the effect of adding BNP or NT-proBNP to this risk score model in a Korean heart failure registry population.

## Methods

### Study population

The Korean Acute Heart Failure (KorAHF) registry is a prospective multicenter cohort study designed to measure the outcomes of Korean patients admitted for HF. The registry data consist of patient demographics, clinical characteristics, and evidence-based treatments[3, 25]. Patients who had signs or symptoms of HF and met one of the following criteria were eligible for this study: (1) lung congestion or (2) objective findings of left ventricular systolic dysfunction or structural heart disease. Patients hospitalized for AHF from 10 tertiary university hospitals throughout the country were consecutively enrolled from March 2011 to February 2014. The patients were planned to be followed up until 2018. Data were collected by each site and entered into a web-based case-report form in the Clinical Data Management System from the Korea National Institute of Health. Information about patient demographics, medical history, signs, symptoms, results of laboratory tests, electrocardiogram, echocardiography, medications, hospital course, and outcomes was collected at admission, discharge, and follow-up (30days, 90days, 180days, 1to 5years annually).Written informed consent was obtained from each patient. If patients were unable to give consent due to disease severity, informed consent was obtained from a relative or legal representative. In-hospital mortality and the mode of death have been adjudicated by an independent event committee. The mortality data for patients lost to follow-up were collected from the national insurance data or national death records. The study protocol was approved by the ethics committee/institutional review board (IRB) at each hospital.

### Measurement of BNP or NT-proBNP

Plasma BNP or NT-proBNP levels were measured at admission for acute HF. Blood sampling and tests were conducted as routine practice by laboratories at each center certified by the Korean Association of Quality Assurance for Clinical Laboratories[26]. Measurement of NT-proBNP was performed with the electro-chemiluminescence immunoassay method using an Elecsys 2010 analyzer (Roche Diagnostics) or NT-proBNP assay for Dimension platform, Siemens Medical Solutions Diagnostics. Plasma was tested for BNP using the Biosite Triage assay, a point-of-care device that uses a fluorescence immunoassay technique (Biosite, San Diego, CA, USA)[27].

### Statistical analysis

Descriptive statistics are used to summarize demographic and clinical characteristics, clinical care during hospitalization, and outcomes of the patients. Continuous variables are presented as mean ± standard deviation (SD) and were analyzed with Student’s *t*-test to demonstrate the statistical significance of the difference between two groups. Categorical variables are presented as frequency (percentage) and were analyzed with Chi-square test. The logistic regression model was applied to verify predictors of all cause mortality. Score discrimination and calibration were evaluated by the c statistic and Hosmer-Lemeshow statistic [9, 11]. To test the incremental prognostic utility of B-type natriuretic peptide level (BNP) or N-terminal pro-B-type natriuretic peptide (NT-proBNP), we used the AUC comparison as DeLoog, net reclassification index (NRI) and integrated discrimination improvement (IDI). P-value less than 0.05 were considered statistically significant. All analyses were performed using SAS statistical software (version 9.4; SAS Institute Inc., Cary, NC).

## Results

### Demographic characteristics and clinical profiles

**Fig 1:**
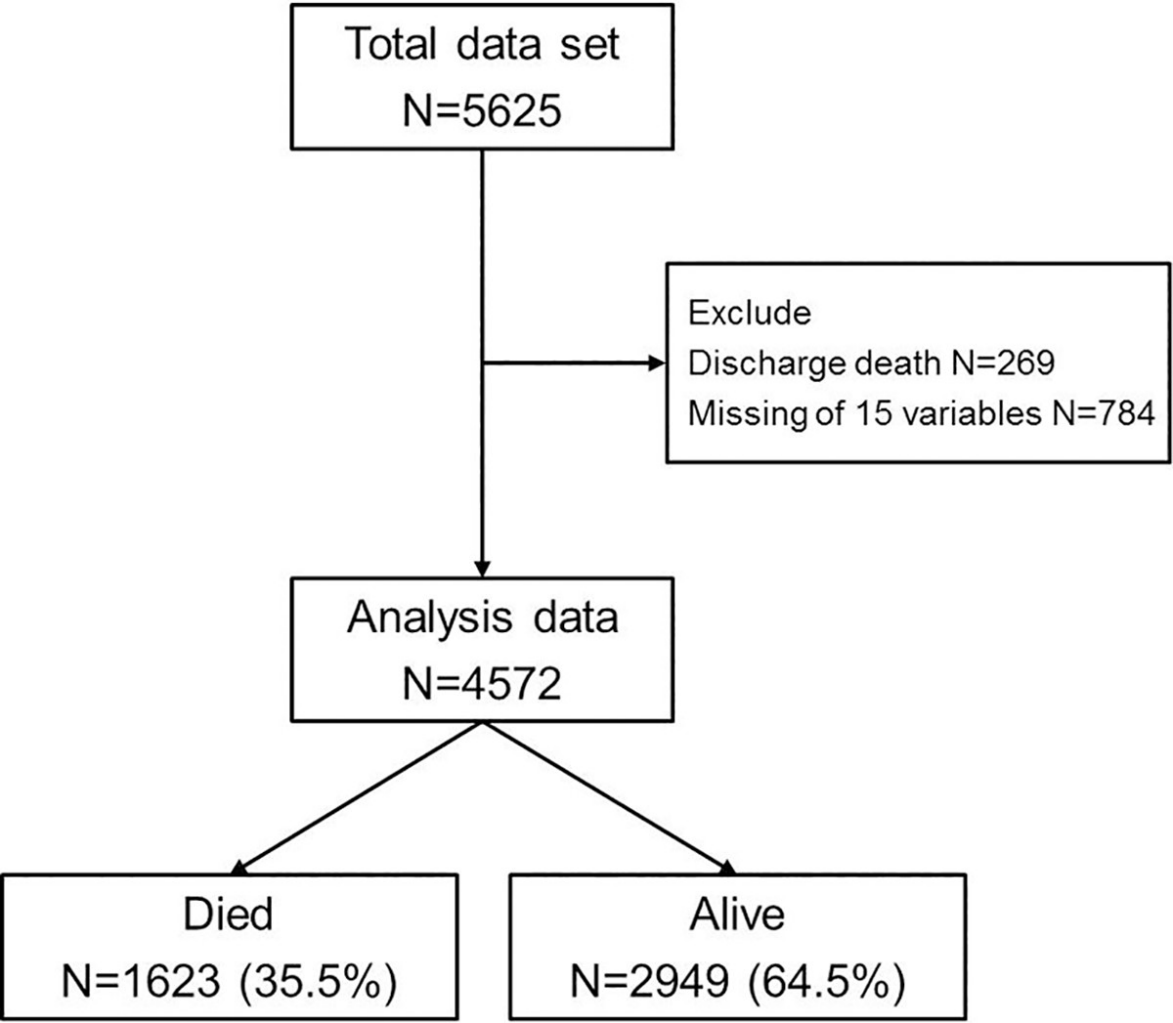
Selection of the study population. This study enrolled 5625 patients. We excluded 269patients who died in hospital, 784 patients with missing patient characteristics, and patients without death information. Finally, we analyzed 4572 patients including 1623 (35.5%) dead and 2949 (64.5%) alive.

We enrolled 5,625 AHF subjects from 10 tertiary university hospitals in Korea. We excluded 269 patients who died in hospital, 784 patients with missing patient characteristics, and patients without death information. Finally, we analyzed 4572 patients including 1623 (35.5%) dead and 2949 (64.5%) alive (Fig 1).

Descriptive statistics for patients who are still alive and patients who died during follow-up are summarized in Table 1.The mean age was68.42±14.52 years, and 2392(52.32%)were men. The overall 1-year mortality in the study population was 17.58%. Among the 13 variables, age, creatinine (Cr), smoker and diabetes (41.53%),COPD(14.17%), HF duration > 18 month (52.37%) and NYHA 4(53.73%) as well as not taking medications (ACEI or ARB and beta blocker) were significantly higher in patients who died during follow-up compared with those who are alive. Additionally, body mass index (BMI)was almost similar between the 2 groups. However, male gender, systolic blood pressure (BP), and ejection fraction did not differ significantly between the two groups. High plasma BNP(>887.9) or NT-proBNP (>4751.05) according to the median value was significantly higher in the mortality group (61.31% vs 44.63%, p<0.0001), whereas low BNP or NT-proBNP was higher in the alive group (38.69% vs 55.37%, p<0.0001).

**Table 1:** Descriptive statistics for baseline variables.

variables			Total	Death	Alive	p-value
			N = 4572	N = 1623(35.5%)	N = 2949 (64.5%)	
age			68.42±14.52	74.30±11.54	65.18±14.97	< .0001
Gender (Male)			2392(52.32%)	783(48.24%)	1397(47.37%)	0.5722
BMI			23.35±3.90	22.41±3.71	23.86±3.91	< .0001
Current smoker	Current smoker		812 (17.76%)	213(13.12%)	599(20.31%)	< .0001
	Ex-smoker		982(21.48%)	386(23.78%)	596(20.21%)	
	Never smoker		2778(60.76%)	1024(63.09%)	1754(59.48%)	
SBP			133.0±30.34	132.3±30.63	133.4±30.17	0.2448
DM			1598 (34.95%)	674(41.53%)	924(31.33%)	< .0001
NYHA	2		688(15.05%)	176(10.84%)	512(17.36%)	< .0001
	3		1675 (36.64%)	575(35.43%)	110(37.30%)	
	4		2209(48.32%)	872(53.73%)	1337(45.34%)	
Ejection fraction			38.37±15.73	38.54±15.73	38.27±15.73	0.5747
COPD			515 (11.26%)	230(14.17%)	285(9.66%)	< .0001
HF duration > 18 month			1924 (42.08%)	850(52.37%)	1074(36.42%)	< .0001
Creatinine (μmol/L)			127.62±121.09	149.2±126.7	115.8±116.2	< .0001
Beta blocker	Yes		2423(53%)	740(45.59%)	1683(57.07%)	< .0001
	No		2149(47%)	883(54.41%)	1226(42.93%)	
ACEI or ARB	Yes		3219(70.41%)	1061(65.37%)	2158(73.18%)	< .0001
	No		1353 (29.59%)	562 (34.63%)	791(26.82%)	
BNP or NTproBNP	< median		2261(49.45%)	628(38.69%)	1633(55.37%)	< .0001
	≥ median		2311(50.55%)	995(61.31%)	1316(44.63%)	

Values are expressed as mean±standard deviation or n (%). BMI, body mass index; DM, diabetes mellitus; HTN, hypertension; HF, heart failure; COPD, chronic obstructive pulmonary disease; SBP, systolic blood pressure; EF, ejection fraction; creatinine;New York Heart Association (NYHA) BNP, brain natriuretic peptide. Angiotensin converting enzyme inhibitors (ACE inhibitors)angiotensin receptor blocker(ARB)

### Predictors of mortality

In multivariable analysis, 11 variable included in the MAGGIC score were associated with mortality. However, when BNP or NT-pro-BNP were associated with MAGGIC. ejection fraction could not predict mortality (Table 2).Here 11variables(age, male sex, BMI,SBP, diabetes, HF duration > 18 months, NYHA(2–4),Cr, ACEI or ARB, and beta blocker, along with high BNP or NT-proBNP) were independent predictors in multivariable analysis(Table 2). However, smoker and COPD were non-significantly associated with mortality in both groups. The risk score was significantly different between alive and dead groups (30.61 ± 6.32 vs. 24.80 ± 6.81, p<0.001). After the conjoint use of BNP or NT-proBNP and MAGGIC risk score in patients with HF, the risk score was also significantly different (31.23 ± 6.46 vs. 25.25 ± 6.96, p<0.001) (Table 3).There were strongly significantly difference between them [0.61 (0.59~0.64) for death group and 0.45 (0.43~0.47) for alive group].

**Table 2:** Multivariate analysis using variables included in the MAGGIC score.

		MAGGIC score			MAGGIC score+BNP or NTproBNP		
variables		OR	95% CI	p-value	OR	95% CI	p-value
age		1.056	1.049~1.062	< .0001	1.055	1.048~1.061	< .0001
Gender (male)		1.28	1.077~1.521	0.005	1.339	1.125~1.573	0.001
BMI		0.925	0.908~0.943	< .0001	0.933	0.915~0.951	< .0001
Current smoker	Current smoker	0.883	0.709~1.099	0.5265	0.87	0.698~1.085	0.4475
	Ex-smoker	0.938	0.771~1.142		0.926	0.760~1.127	
	Never smoker	1	-		1	-	
SBP		0.995	0.993~0.997	< .0001	0.995	0.992~0.997	< .0001
DM		1.481	1.286~1.704	< .0001	1.474	1.280~1.698	< .0001
NYHA	2	1	-	0.0018	1	-	0.0108
	3	1.341	1.080~1.665		1.301	1.047~1.618	
	4	1.464	1.186~1.806		1.385	1.120~1.712	
Ejection fraction		0.995	0.990~1.000	0.033	0.998	0.993~1.003	0.4067
COPD		1.124	0.919~1.376	0.2555	1.149	0.938~1.407	0.1789
HF duration > 18 month		1.583	1.381~1.813	< .0001	1.564	1.364~1.793	< .0001
Creatinine		1.002	1.002~1.003	< .0001	1.002	1.001~1.002	< .0001
Beta blocker	Yes	1	-	< .0001	1	-	< .0001
	No	1.394	1.216~1.597		1.385	1.207~1.588	
ACEI or ARB	Yes	1	-	0.0006	1	-	0.0006
	No	1.3	1.120~1.508		1.299	1.119~1.508	
BNP or NTproBNP	< median	-	-	-	1	-	< .0001
	≥ median				1.476	1.276~1.708	

Values are expressed as mean±standard deviation or n (%). BMI, body mass index; DM, diabetes mellitus; HTN, hypertension; HF, heart failure; COPD, chronic obstructive pulmonary disease; SBP, systolic blood pressure; EF, ejection fraction; creatinine;New York Heart Association (NYHA) BNP, brain natriuretic peptide. Angiotensin converting enzyme inhibitors (ACE inhibitors)angiotensin receptor blocker (ARB)

**Table 3:** Risk score according to death.

	Death	Alive	p-value
	N = 1623 (35.5%)	N = 2949 (64.5%)	
Maggic score	30.61 ± 6.32	24.80 ± 6.81	< .0001
Maggicscore+BNP or NTproBNP	31.23 ± 6.46	25.25 ± 6.96	< .0001
Difference (95%CI)	0.61 (0.59~0.64)	0.45 (0.43~0.47)	< .0001
Withinp-value	< .0001	< .0001	

### Validation of the risk score models

**Fig 2:**
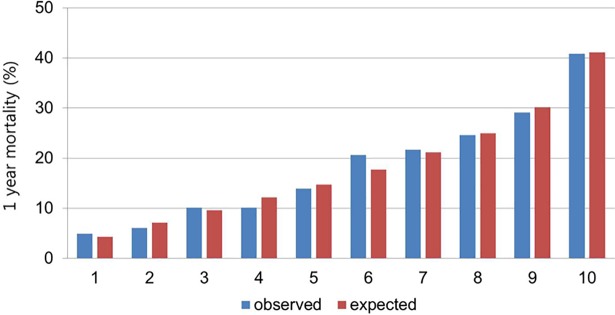
Observed vs.Model-predicted 1-year mortality in risk groups: Hosmer-Lemeshow goodness of fit test for risk score mortality indicated a good calibration (p = 0.5559), the predicted mortality rates corresponded with the observed mortality rates in each decile.

In this study, we selected 13 predictor variables from the multivariable model to formulate a risk score. Good discrimination abilities were found,with a C-indexof 0.734 from the risk score model to predict 1-year mortality. Hosmer-Lemeshow goodness of fit test for risk score mortality indicated a good calibration (p = 0.5559) by plotting predicted versus observed mortality using 10 groups (Fig 2). Additionally, the predicted mortality rates corresponded with the observed mortality rates in each decile.

### Effect of adding natriuretic peptide

The addition of BNP or NT-proBNP to the MAGGIC risk score using the 13 variables resulted in minimal improvement(C index of 0.734 for MAGGIC risk score and 0.736 for MAGGIC risk score plus BNP or NT-proBNP, p = 0.0502).However, we achieved a significant improvement in net reclassification and integrated discrimination for mortality (NRI of 33.4%,p <0.0001 and IDI of 0.002, p <0.0001) (Table 4).

**Table 4:** Net reclassification improvement (NRI) and integrated discrimination improvement (IDI) for specific models using the BNP.

	Values	p-value
**C-index (95%CI)**		
Maggic score	0.734 (0.720~0.749)	-
Maggicscore+BNP or NTproBNP	0.736 (0.721~0.750)	0.0502
**NRI (95% CI)**		
Category-free NRI (%)	33.4% (27.4%~39.3%)	< .0001
% of events correctly reclassified	23%	
% of non-events correctly reclassified	11%	
**IDI (95% CI)**	0.002 (0.001~0.003)	< .0001

## Discussion

This study demonstrates the performance of the MAGGIC project HF risk score using 13 variables by external validation to predict 1-year mortality after discharge in Korean hospitalized patients with HF. We found that the discrimination and calibration were good for the MAGGIC risk score. This risk score is useful to predict mortality after discharge for hospitalized patients with HF. Furthermore, the addition of BNP or NT-proBNP to the MAGGIC risk score showed significant improvement in risk reclassification of patients and minimal improvement in discrimination ability.

HF is a complex and fatal medical condition that progresses rapidly with escalating aging population and causes considerable morbidity, mortality, and re-hospitalization, resulting in a tremendous burden on the global healthcare system [28–30]. Physicians always need to determine the best therapy for high-risk patients and make estimation of risk in patients by integrating patient characteristics, clinical signs, and laboratory tests[8].Prediction is naturally inconsistent. Although a physician assigns a relative weight to each variable, which relies on his previous experiences, personal beliefs, clinical judgment, as well as current mood, it could be inaccurate and ambiguous. To determine an individual’s cardiac mortality risk score in daily clinical practice, adding points assigned for predictors that exist in a patient is only required. For this reason, the MAGGIC risk score is beneficial for busy clinicians and nurses. Using the 13 available baseline variables, including age, EF, NYHA class, serum Cr, diabetes, systolic BP, BMI, HF duration, current smoker, COPD, male gender, evidence-based medication beta blocker, and ACE inhibitor or ARBs, can construct the risk score and identify the risks of hospitalized patients easily at low cost. In this MAGGIC score, each variable was independently associated with mortality, except COPD and smoker.

Several risk stratification models that are used to predict mortality in hospitalized patients with HF have limitations. For example, the Seattle Heart Failure Model and the CHARM-model use not readily available variables. The former requires lymphocyte count, uric acid, and total cholesterol, and the latter requires atrial fibrillation, cardiomegaly, and pulmonary edema. Meanwhile, in the HF-ACTION predictive risk score model, inclusion of exercise duration on the baseline cardiopulmonary exercise test and serum urea nitrogen, which require difficult estimations, is necessary[31–33].Moreover, in assessing long-term mortality, other predictive models are used, but included participants from clinical trials. The models also rely on not readily available variables in clinical care, such as peak exercise oxygen consumption for the Heart Failure Survival Score model; and hemoglobin, GGT, eGFR, 12-lead ECG, and 24-h Holter monitoring variables including non-sustained VT and frequent VPBs,for the MUSIC risk score model[34, 35]. Although these predictive models are informative and important for research purposes, their drawbacks limit their use in daily clinical practice.By contrast, the MAGGIC project HF risk score has several advantages and is superior to other HF mortality risk models. Itis targeted for a great number of population-based patients with manifold demographic characteristics regardless of LV systolic function and can be used for nationwide contemporary external validation cohort. Thus, this model is extensively pertinent and appealing as it contains nearly a modest number of variables regularly collected during admission[9]. MAGGIC risk score can be calculated by entering admission data into an online risk calculator (www.heartfailurerisk.org).This calculator can estimate1-year all-cause mortality for patients with HF. It is not only easily understandable and beneficial to physicians, but also helpful for patients and relatives[11].

The MAGGIC projectincluded39,372 patients from 30 studies. Of these studies, 6 were randomized controlled trials (24,041 patients) and the remaining 24 were registries (15,331 patients).Approximately 40% of patients died during a median follow-up of 2.5 years.The six largest studiescontributed76% of patients and 76% of deaths [11]. Recently, another study performed the MAGGIC risk score in a Swedish population and found excellent discrimination. However, good calibration was not found as moderate increase in low-risk patients and depreciation of high-risk patients accounted for the 3-year mortality risk[9]. In this present study, we observed good discrimination and calibration for MAGGIC risk score in hospitalized patients with HF derived from the KorAHF after discharge. Discharge is often the first step of treatment progress and initial assessment for prediction of prognosis. We adopted the risk score to patients with HF after discharge from hospital.

This study is the first to use the MAGGIC risk score of patients with HFin a population in Korea, which is part of East Asia. Some differences exist in the baseline clinical characteristics and co-morbidity between Western and East Asian countries[36].Several studies reported that the analytical histories of IHD and hypertension in Koreawere45.4% and 43.6%, respectively, which were considerably lower than in the high-income Western nations[37]. On the contrary, the prevalence of hypertension as a co-morbidity of patients with HF is widely varied (39.2% to 77.6%) in Japan than in other studies, such as in the Acute Decompensated Heart Failure Syndromes registry (71%),the Organized Program to Initiate Lifesaving Treatment in Hospitalized Patients with Heart Failure (71%),and the Euro-Heart Failure Survey II (63%)[38–41].Although the prevalence of HF is lower in Korea (1.53%) compared with that in the USA (2.2%) or other Western and European nations, the increase in aging population and adoption of a Western life style promote the growth of HF prevalence in East Asian countries, including Korea, China, and Japan[37, 42].With gender consideration, male is the most classic demographic risk factor for the development of HF, along with older age, ethnicity, and low socio-economic state, in East Asia [43].The prevalence of overweight/obesity is much lower(21.6–26.2%) in Southeast Asia than in the UK (66.7%) and USA (69.6%). However, the recent prevalence of blood glucose/diabetes as a notable risk factor is higher in Asian countries than in the UK and USA[44].During the follow-up period, the clinical effect of evidenced-based medication between Western and Asian countries was also different[45]. Therefore, establishment of MAGGIC HF risk score model is not only important for Korean population but also for other Asian countries.

BNP and NT-proBNP are the most powerful prognostic factors; they are expected to serve as an important basis for diagnosis and play a crucial role in therapeutic decision-making process[22]. High plasma BNP or NT-proBNP at admission predicts greater risk of outcomes in hospitalized patients with HF[22, 46]. On the contrary, we added BNP or NT-proBNP to the MAGGIC risk score and found minimal improvement in discrimination (C index of 0.734 for MAGGIC risk score and 0.736 for MAGGIC risk score plus BNP or NT-proBNP, p = 0.0502). In multivariable analysis, we observed that high natriuretic peptide level was significantly associated with mortality. Furthermore, when we used new statistical analysis methods (NRI and IDI indices), BNP or NT-proBNP (NRI of 33.4%, p <0.0001 and IDI of 0.002, p <0.0001)showed the greatest increase in discrimination and net reclassification for mortality. Based on this study, we suggest that the validated risk score is readily generalizable, and the addition of BNP or NT-proBNPto the MAGGIC risk score was beneficial in predicting more death. Therefore, a quick and cost-effective important indicator is established to anticipate adverse events in patients with HF. This indicator supports the performing cardiologist and referring clinician to take direct care and improve communication with patients with HF about their prognosis[46].

## Study limitations

This cohort study has several limitations. First, its design had several inherent limitations including selection bias and missing values. Second, we did not use MAGGIC risk score in-hospital patients with unstable health condition during admission. Thus, we excluded in-hospital mortality, and analysis was considered after discharge of patients. Third, although in-hospital mortality and post-discharge mortality were confirmed by an independent event committee, causes of post-discharge mortality are unknown. We could not differentiate cardiovascular mortality and causes of re-hospitalization, which were not validated. Fourth, we did not perform the specific categories of BNP or NTproBNP levels to be widely used as a prognostic score. We could not also validate and compare MAGGIC risk score with other risk score such as 3C-HF score which is widely used to predict 1-year mortality using routinely available predictors. Finally, we evaluated short-term mortality (1-year mortality), and the biomarker natriuretic peptide(BNP or NT-proBNP)was only used in this risk score. The measurement time and median value of BNP and NT-proBNP were different.

## Conclusions

In the KorAHF, the MAGGIC project HF risk score performed well in a large nationwide contemporary external validation cohort. Furthermore, addition of BNP or NT-proBNPto the MAGGIC risk score was beneficial in predicting more death in hospitalized patients with HF.

## References

[1] Bosseau C, Galli E, Donal E. Prognostic value of BNP in heart failure with preserved or reduced ejection fraction. Heart (British Cardiac Society). 2015;101(23):1855–6. 26363044 10.1136/heartjnl-2015-308515

[2] Logeart DT, Jourdain G., Chavelas P., Beyne C., Beauvais P., Bouvier F., et al Predischarge B-type natriuretic peptide assay for identifying patients at high risk of re-admission after decompensated heart failure. Journal of the American College of Cardiology. 2004;43(4):635–41. 10.1016/j.jacc.2003.09.044 14975475 10.1016/j.jacc.2003.09.044

[3] Lee SE LH, Cho HJ, Choe WS, Kim H, Choi JO, Jeon ES, et al Clinical profiles, management and outcome of acute heart failure in Korea: results from the Korean Acute Heart Failure Registry (KorAHF). Korean Circ J 2017 5;47(3):341–353. 10.4070/kcj.2016.0419 28567084 10.4070/kcj.2016.0419 PMC5449528

[4] Lee SE, Cho HJ, Lee HY, Yang HM, Choi JO, Jeon ES, et al A multicentre cohort study of acute heart failure syndromes in Korea: rationale, design, and interim observations of the Korean Acute Heart Failure (KorAHF) registry. European journal of heart failure. 2014;16(6):700–8. 10.1002/ejhf.91 24797348 10.1002/ejhf.91

[5] Stewart S, MacIntyre K, Hole DJ, Capewell S, McMurray JJ. More 'malignant' than cancer? Five-year survival following a first admission for heart failure. European journal of heart failure. 2001;3(3):315–22. 11378002 10.1016/s1388-9842(00)00141-0

[6] Noveanu M, Breidthardt T, Potocki M, Reichlin T, Twerenbold R, Uthoff H, et al Direct comparison of serial B-type natriuretic peptide and NT-proBNP levels for prediction of short- and long-term outcome in acute decompensated heart failure. Critical care (London, England). 2011;15(1):R1. 10.1186/cc9398 PMC3222028 21208408

[7] Peterson PN, Rumsfeld JS, Liang L, Albert NM, Hernandez AF, Peterson ED, et al A Validated Risk Score for In-Hospital Mortality in Patients With Heart Failure From the American Heart Association Get With the Guidelines Program. Circulation: Cardiovascular Quality and Outcomes. 2009;3(1):25–32. 10.1161/CIRCOUTCOMES.109.854877 20123668 10.1161/CIRCOUTCOMES.109.854877

[8] Passantino A, Monitillo F, Iacoviello M, Scrutinio D. Predicting mortality in patients with acute heart failure: Role of risk scores. World journal of cardiology. 2015;7(12):902–11. 10.4330/wjc.v7.i12.902 26730296 10.4330/wjc.v7.i12.902 PMC4691817

[9] Sartipy U, Dahlstrom U, Edner M, Lund LH. Predicting survival in heart failure: validation of the MAGGIC heart failure risk score in 51,043 patients from the Swedish heart failure registry. European journal of heart failure. 2014;16(2):173–9. 10.1111/ejhf.32 24464911 10.1111/ejhf.32

[10] Somaratne JB, Berry C, McMurray JJ, Poppe KK, Doughty RN, Whalley GA. The prognostic significance of heart failure with preserved left ventricular ejection fraction: a literature-based meta-analysis. European journal of heart failure. 2009;11(9):855–62. 10.1093/eurjhf/hfp103 19654140 10.1093/eurjhf/hfp103

[11] Pocock SJ AC, McMurray JJ, Maggioni A, Kober L, Squire IB, Swedberg K, Dobson J, Poppe KK, Whalley GA, Doughty RN. Predicting survival in heart failure: a risk score based on 39 372 patients from 30 studies. European Heart Journal (2013) 34, 1404–1413. 10.1093/eurheartj/ehs337 23095984 10.1093/eurheartj/ehs337

[12] Ketchum ES, Levy WC. Multivariate risk scores and patient outcomes in advanced heart failure. Congestive heart failure. 2011;17(5):205–12. 10.1111/j.1751-7133.2011.00241.x 21906244 10.1111/j.1751-7133.2011.00241.x

[13] Rusinaru D, Tribouilloy C, Berry C, Richards AM, Whalley GA, Earle N, et al Relationship of serum sodium concentration to mortality in a wide spectrum of heart failure patients with preserved and with reduced ejection fraction: an individual patient data meta-analysis(dagger): Meta-Analysis Global Group in Chronic heart failure (MAGGIC). European journal of heart failure. 2012;14(10):1139–46. 10.1093/eurjhf/hfs099 22782968 10.1093/eurjhf/hfs099

[14] Martinez-Selles M, Doughty RN, Poppe K, Whalley GA, Earle N, Tribouilloy C, et al Gender and survival in patients with heart failure: interactions with diabetes and aetiology. Results from the MAGGIC individual patient meta-analysis. European journal of heart failure. 2012;14(5):473–9. 10.1093/eurjhf/hfs026 22402958 10.1093/eurjhf/hfs026

[15] Berry C DR, Granger C, Køber L, Massie B, McAlister F, McMurray J, et al The survival of patients with heart failure with preserved or reduced left ventricular ejection fraction: an individual patient data meta-analysis. European heart journal. 2012; 33(14):1750–7. 10.1093/eurheartj/ehr254 21821849 10.1093/eurheartj/ehr254

[16] Fonarow GC, Adams KF Jr., Abraham WT, Yancy CW, Boscardin WJ. Risk stratification for in-hospital mortality in acutely decompensated heart failure: classification and regression tree analysis. Jama. 2005;293(5):572–80. 10.1001/jama.293.5.572 15687312 10.1001/jama.293.5.572

[17] Spinar J, Parenica J, Vitovec J, Widimsky P, Linhart A, Fedorco M, et al Baseline characteristics and hospital mortality in the Acute Heart Failure Database (AHEAD) Main registry. Critical care (London, England). 2011;15(6):R291. 10.1186/cc10584 PMC3388663 22152228

[18] Braunwald E. Biomarkers in heart failure. The New England journal of medicine. 2008;358(20):2148–59. 10.1056/NEJMra0800239 18480207 10.1056/NEJMra0800239

[19] Wei BQ, Yang YJ, Zhang J, Dou KF, Zhang YH, Huang XH, et al [Predictive value of admission amino-terminal pro-B-type natriuretic peptide on in-hospital mortality in patients with decompensated heart failure]. Zhonghua xin xue guan bing za zhi. 2009;37(6):481–5. 19927625

[20] Peterson PN, Rumsfeld JS, Liang L, Albert NM, Hernandez AF, Peterson ED, et al A validated risk score for in-hospital mortality in patients with heart failure from the American Heart Association get with the guidelines program. Circulation Cardiovascular quality and outcomes. 2010;3(1):25–32. 10.1161/CIRCOUTCOMES.109.854877 20123668 10.1161/CIRCOUTCOMES.109.854877

[21] Gluba A, Bielecka-Dabrowa A, Mikhailidis DP, Wong ND, Franklin SS, Rysz J, et al An update on biomarkers of heart failure in hypertensive patients. Journal of hypertension. 2012;30(9):1681–9. 10.1097/HJH.0b013e3283569a9c 22828089 10.1097/HJH.0b013e3283569a9c

[22] Huang YT, Tseng YT, Chu TW, Chen J, Lai MY, Tang WR, et al N-terminal pro b-type natriuretic peptide (NT-pro-BNP) -based score can predict in-hospital mortality in patients with heart failure. Scientific reports. 2016;6:29590 10.1038/srep29590 27411951 10.1038/srep29590 PMC4944149

[23] Altman DG, Royston P. What do we mean by validating a prognostic model? Statistics in medicine. 2000;19(4):453–73. 10694730 10.1002/(sici)1097-0258(20000229)19:4<453::aid-sim350>3.0.co;2-5

[24] Kang SH, Park JJ, Choi DJ, Yoon CH, Oh IY, Kang SM, et al Prognostic value of NT-proBNP in heart failure with preserved versus reduced EF. Heart (British Cardiac Society). 2015;101(23):1881–8. 26319121 10.1136/heartjnl-2015-307782

[25] Choi H, Yoo BS, Doh JH, Yooh HJ, Ahn MS, Kim JY, et al The optimal time of B-type natriuretic peptide sampling associated with post-myocardial infarction remodelling after primary percutaneous coronary intervention. Cardiovascular journal of Africa. 2013;24(5):165–70. 10.5830/CVJA-2013-024 24217163 10.5830/CVJA-2013-024 PMC3748455

[26] McMurray JJ, Stewart S. Epidemiology, aetiology, and prognosis of heart failure. Heart (British Cardiac Society). 2000;83(5):596–602. 10768918 10.1136/heart.83.5.596 PMC1760825

[27] Chen J, Normand SL, Wang Y, Krumholz HM. National and regional trends in heart failure hospitalization and mortality rates for Medicare beneficiaries, 1998–2008. Jama. 2011;306(15):1669–78. 10.1001/jama.2011.1474 22009099 10.1001/jama.2011.1474 PMC3688069

[28] Santas E, de la Espriella-Juan R, Mollar A, Valero E, Minana G, Sanchis J, et al Echocardiographic pulmonary artery pressure estimation and heart failure rehospitalization burden in patients with acute heart failure. International journal of cardiology. 2017. 10.1016/j.ijcard.2017.04.055 28455131

[29] Pocock SJ, Wang D, Pfeffer MA, Yusuf S, McMurray JJ, Swedberg KB, et al Predictors of mortality and morbidity in patients with chronic heart failure. European heart journal. 2006;27(1):65–75. 10.1093/eurheartj/ehi555 16219658 10.1093/eurheartj/ehi555

[30] Levy WC, Mozaffarian D, Linker DT, Sutradhar SC, Anker SD, Cropp AB, et al The Seattle Heart Failure Model: prediction of survival in heart failure. Circulation. 2006;113(11):1424–33. 10.1161/CIRCULATIONAHA.105.584102 16534009 10.1161/CIRCULATIONAHA.105.584102

[31] O'Connor CM, Whellan DJ, Wojdyla D, Leifer E, Clare RM, Ellis SJ, et al Factors related to morbidity and mortality in patients with chronic heart failure with systolic dysfunction: the HF-ACTION predictive risk score model. Circulation Heart failure. 2012;5(1):63–71. 10.1161/CIRCHEARTFAILURE.111.963462 22114101 10.1161/CIRCHEARTFAILURE.111.963462 PMC3692371

[32] Aaronson KD, Schwartz JS, Chen TM, Wong KL, Goin JE, Mancini DM. Development and prospective validation of a clinical index to predict survival in ambulatory patients referred for cardiac transplant evaluation. Circulation. 1997;95(12):2660–7. 9193435 10.1161/01.cir.95.12.2660

[33] Vazquez R, Bayes-Genis A, Cygankiewicz I, Pascual-Figal D, Grigorian-Shamagian L, Pavon R, et al The MUSIC Risk score: a simple method for predicting mortality in ambulatory patients with chronic heart failure. European heart journal. 2009;30(9):1088–96. 10.1093/eurheartj/ehp032 19240065 10.1093/eurheartj/ehp032

[34] Rusinaru D TC, Berry C, Richards AM, Whalley GA, Earle N, Poppe KK, et al MAGGIC Investigators. Relationship of serum sodium concentration to mortality in a wide spectrum of heart failure patients with preserved and with reduced ejection fraction: an individual patient data meta-analysis(†): Meta-Analysis Global Group in Chronic heart failure (MAGGIC). Eur J Heart Fail 2012 10;14(10):1139–46. 10.1093/eurjhf/hfs099 22782968 10.1093/eurjhf/hfs099

[35] Lee JH, Lim NK, Cho MC, Park HY. Epidemiology of Heart Failure in Korea: Present and Future. Korean circulation journal. 2016;46(5):658–64. 10.4070/kcj.2016.46.5.658 27721857 10.4070/kcj.2016.46.5.658 PMC5054178

[36] Mosterd A, Hoes AW. Clinical epidemiology of heart failure. Heart (British Cardiac Society). 2007;93(9):1137–46. 17699180 10.1136/hrt.2003.025270 PMC1955040

[37] Kajimoto K, Sato N, Keida T, Mizuno M, Sakata Y, Asai K, et al Association between length of stay, frequency of in-hospital death, and causes of death in Japanese patients with acute heart failure syndromes. International journal of cardiology. 2013;168(1):554–6. 10.1016/j.ijcard.2013.01.187 23434007 10.1016/j.ijcard.2013.01.187

[38] Abraham WT, Fonarow GC, Albert NM, Stough WG, Gheorghiade M, Greenberg BH, et al Predictors of in-hospital mortality in patients hospitalized for heart failure: insights from the Organized Program to Initiate Lifesaving Treatment in Hospitalized Patients with Heart Failure (OPTIMIZE-HF). Journal of the American College of Cardiology. 2008;52(5):347–56. 10.1016/j.jacc.2008.04.028 18652942 10.1016/j.jacc.2008.04.028

[39] Nieminen MS, Brutsaert D, Dickstein K, Drexler H, Follath F, Harjola VP, et al EuroHeart Failure Survey II (EHFS II): a survey on hospitalized acute heart failure patients: description of population. European heart journal. 2006;27(22):2725–36. 10.1093/eurheartj/ehl193 17000631 10.1093/eurheartj/ehl193

[40] Okamoto H, Kitabatake A. [The epidemiology of heart failure in Japan]. Nihon rinsho Japanese journal of clinical medicine. 2003;61(5):709–14. 12754992

[41] Schocken DD, Benjamin EJ, Fonarow GC, Krumholz HM, Levy D, Mensah GA, et al Prevention of heart failure: a scientific statement from the American Heart Association Councils on Epidemiology and Prevention, Clinical Cardiology, Cardiovascular Nursing, and High Blood Pressure Research; Quality of Care and Outcomes Research Interdisciplinary Working Group; and Functional Genomics and Translational Biology Interdisciplinary Working Group. Circulation. 2008;117(19):2544–65. 10.1161/CIRCULATIONAHA.107.188965 18391114 10.1161/CIRCULATIONAHA.107.188965

[42] Lam CSP. Heart failure in Southeast Asia: facts and numbers. ESC Heart Failure. 2015;2(2):46–9. 10.1002/ehf2.12036 28834655 10.1002/ehf2.12036 PMC6410537

[43] Yoo BS, Oh J, Hong BK, Shin DH, Bae JH, Yang DH, et al SUrvey of Guideline Adherence for Treatment of Systolic Heart Failure in Real World (SUGAR): a multi-center, retrospective, observational study. PloS one. 2014;9(1):e86596 10.1371/journal.pone.0086596 24475154 10.1371/journal.pone.0086596 PMC3903552

[44] Santaguida PL, Don-Wauchope AC, Oremus M, McKelvie R, Ali U, Hill SA, et al BNP and NT-proBNP as prognostic markers in persons with acute decompensated heart failure: a systematic review. Heart failure reviews. 2014;19(4):453–70. 10.1007/s10741-014-9442-y 25062653 10.1007/s10741-014-9442-y

[45] Ahn MS and Yoo BS. Serial Monitoring of B-Type Natriuretic Peptide in Heart Failure Patients. Korean Circulation J 2007 2007;37: p 393–398.

[46] Denardo SJ, Vock DM, Schmalfuss CM, Young GD, Tcheng JE, O'Connor CM.Baseline Hemodynamics and Response to Contrast Media During Diagnostic Cardiac Catheterization Predict Adverse Events in Heart Failure Patients. CirculationHeart failure. 2016;9(7). 10.1161/CIRCHEARTFAILURE.115.002529 27382090

